# Sociodemographic and Dog‐Management Factors Associated With Free‐Roaming Dogs in Nigeria and Sierra Leone

**DOI:** 10.1155/tbed/8621173

**Published:** 2026-09-29

**Authors:** Philip P. Mshelbwala, J. Scott Weese, Tasneem Imam, Harish K. Tiwari, Roland Suluku, Charles E. Rupprecht

**Affiliations:** ^1^ Department of Primary Industries and Regional Development Orange New South Wales Australia; ^2^ School of Veterinary Sciences University of Queensland Gatton Australia uq.edu.au; ^3^ Department of Pathobiology University of Guelph Ontario Canada uoguelph.ca; ^4^ Jyoti and Bhupat Mehta School of Health Science and Technology Indian Institute of Technology Guwahati Guwahati Assam India iitg.ac.in; ^5^ Njala University Bo Sierra Leone njala.edu.sl; ^6^ College of Forestry, Wildlife and Environment College of Veterinary Medicine Auburn University Auburn Alabama USA auburn.edu

## Abstract

Free‐roaming dogs (FRDs) pose a persistent challenge to canine rabies elimination in low‐ and middle‐income countries, as conventional vaccination strategies often struggle to reach this population, which increases the risk of dog bites, rabies virus exposure and traffic accidents and complicates rabies control efforts. We conducted cross‐sectional surveys in Nigeria and Sierra Leone and fitted country‐specific probabilistic models to identify factors associated with FRD ownership. The proportion of FRDs was higher in Sierra Leone (78.7%; 95% CI: 77.1–80.3) than in Nigeria (58.5%; 95% CI: 58.2–61.2). In both countries, significant interactions between locality (urban versus rural), education level, access to veterinary services and dog vaccination status influenced FRD ownership. The probability of FRD ownership was higher among respondents with no formal education, particularly those living in rural areas with no access, or uncertain access, to veterinary services. In Nigeria, FRD ownership increased with household poverty. In Sierra Leone, it was highest in low‐density rural areas, declined sharply with increasing population density and decreased with distance from roads. The interaction between literacy and primary education was associated with reduced FRD ownership, although neither variable was significant when considered independently. In both countries, owners with good rabies knowledge and owners of indigenous, rather than mixed‐breed, dogs were more likely to own FRDs. Spatial variation in FRD ownership was evident in both countries. In Nigeria, dog owners in Kaduna and the Federal Capital Territory (FCT) were less likely to own FRDs than those in Anambra and Enugu. In Sierra Leone, the probability of FRD ownership was substantially higher in northern than in southern regions. Based on human‐to‐dog ratios of 2.77:1 in Nigeria and 2.75:1 in Sierra Leone, we estimated dog populations of ~11.7–12.7 million and 666,000, respectively. These findings highlight the need for targeted public education on responsible dog ownership and effective dog management, alongside community‐level interventions in rural and other high‐risk areas to reduce FRD populations. The resulting population estimates provide valuable evidence for planning and implementing mass canine rabies vaccination programs in both countries.

## 1. Introduction

Free‐roaming dogs (FRDs), comprising owned dogs allowed to roam, stray dogs, feral dogs and community dogs, constitute ~75% of the estimated 700 million dogs worldwide [1–4]. In most low‐ and middle‐income countries (LMICs), FRDs pose a substantial public health burden through zoonotic disease transmission, dog‐bite injuries, traffic accidents and environmental contamination of soil and water with pathogens, particularly in densely populated urban and peri‐urban communities [5–9]. Unrestricted roaming facilitates uncontrolled mating. Combined with high fertility rates and limited access to sterilisation services, this drives rapid population growth and perpetuates the expansion of FRD populations [5, 6]. A critical challenge in controlling FRDs is that they represent a hard‐to‐reach population for parenteral vaccine delivery. Their large population size may hinder the success of rabies elimination programs that rely solely on parenteral vaccination, making oral rabies vaccination a promising complementary strategy [7]. Measures aimed at controlling FRDs and reducing associated disease risks must be underpinned by an understanding of the factors that influence owners to allow their dogs to roam freely. Such knowledge is essential for developing evidence‐based strategies to improve vaccine access and coverage. In Nigeria, Africa’s most populous country, most FRDs are owned yet remain inadequately vaccinated or unvaccinated against rabies, posing a major obstacle to rabies elimination efforts [8]. Similarly, in Freetown, Sierra Leone, the population of FRDs increased markedly following the civil war and the Ebola virus disease epidemic, resulting in large numbers of abandoned and unmanaged dogs roaming urban and recreational areas [9, 10]. Rising dog populations in these contexts are accompanied by increasing reports of bites, persistent viral transmission and growing public health concerns. Inadequate vaccination coverage further heightens the risk of sustained viral circulation [10–12]. Despite these challenges, existing research in both countries has primarily focused on the dog population structure and vaccination coverage [13–16], whereas the determinants of FRD ownership and free‐roaming behaviour remain poorly understood. Little attention has been given to the behavioural and socio‐cultural factors influencing owners’ decisions to allow their dogs to roam freely. This knowledge gap limits the development of targeted, evidence‐based interventions. Our study aimed to identify sociodemographic and dog ownership factors associated with the ownership of FRDs in Nigeria and Sierra Leone, to inform more effective dog management and rabies control strategies.

## 2. Methods

### 2.1. Ethical Approval

Ethical approval was obtained from the Directorate of Livestock and Veterinary Services, Ministry of Agriculture and Food Security, Sierra Leone, and the University of Abuja Ethics Committee on Animal Use (UAECAU/2023/004), as well as the Federal Capital Territory (FCT) Health Research Ethics Committee, Nigeria (/FHREC/2019/01/04/21–01‐19). Verbal informed consent was obtained from all participants before inclusion in the study.

### 2.2. Study Design

This study builds upon previously described cross‐sectional household surveys conducted independently in Sierra Leone and Nigeria. Detailed protocols have been published elsewhere [11, 17]. Briefly, in Sierra Leone, data were collected across the Eastern, Southern, Northern–Western and Western Area provinces. In Nigeria, the study was conducted in selected high‐risk rabies locations in Kaduna State (northwest), the FCT (northcentral) and Enugu and Anambra States (southeast). Site selection in Nigeria was guided by national rabies surveillance data and published reports indicating a higher canine rabies burden in these regions, while in Sierra Leone, settlements were selected based on reports of dog bites and cat bites [10, 18].

A multistage cross‐sectional sampling design was used in both countries. In the selected areas, systematic random sampling was used to identify households. Streets were first selected, and within each selected street, every tenth household was approached for an interview. In locations without formal street layouts, households were identified using community guidance or local markers [14]. Households without dogs or those that declined participation were skipped for the next eligible household. Interviews were conducted with an adult household member.

### 2.3. Questionnaire Administration

The questionnaire instrument and administration procedures were previously described in detail [11, 17]. Briefly, trained enumerators conducted face‐to‐face interviews using structured questionnaires administered via handheld electronic devices. The questionnaire was developed in English and translated, where necessary, into the locally spoken languages. In Sierra Leone, interviews were conducted in English, Krio, Mende, Temne or Limba. In Nigeria, translations were provided in Hausa, Igbo and Yoruba to ensure inclusivity.

### 2.4. Data Collected

The questionnaire covered three main areas: first, sociodemographic characteristics of dog owners, including sex, education, occupation, household size and number of dogs; second, management practices, including the primary purpose of dog ownership.Confinement was measured by asking: ‘Is your dog under confinement?’ with response options of ‘yes’, ‘no’ or ‘partial confinement’. Responses were subsequently reclassified as confined versus unconfined; access to veterinary services (yes, no and not aware) and vaccination status. Third, knowledge and practices related to rabies, including familiarity with the disease, viral transmission routes, susceptible hosts, history of dog bites and intended health‐seeking behaviour following a dog bite. For unvaccinated dogs, follow‐up questions assessed reasons for non‐vaccination (e.g., cost, access and perceived importance).

We obtained additional spatial covariates previously reported as influencing FRD ownership [19], including a poverty raster map representing the proportion of individuals living below the poverty line per grid cell across Nigeria at ~1 km resolution [20] and a literacy raster map showing the proportion of men and women aged 15–49 per grid square as literate in Nigeria at ~1 km resolution [21]. We also obtained the distance (in km) to the nearest road using OpenStreetMap. To characterise settlement patterns, we used the Urban Extent Grid (v1), which provides the proportion of rural and urban areas per grid cell, derived from the Global Rural–Urban Mapping Project (GRUMP v1) [22]. For Sierra Leone, we obtained population estimates from a gridded population raster, with values extracted at the geographic coordinates of each surveyed household (https://diva-gis.org/data.html). The raster had a spatial resolution of ~730 m, and the extracted values represent the estimated population within the corresponding grid cell. For all the rasters, household geographic coordinates were converted to spatial point objects and projected to the same coordinate reference system (WGS84) rasters before extraction using the raster package Version 3.6.32 [23]. The extracted values were subsequently used as a covariate in the statistical analyses.

### 2.5. Estimation of Dog Population

For each country, the total number of dogs in the sample was calculated by summing the product of the number of dogs per household and their observed frequencies. Similarly, the total number of humans was calculated using the reported household sizes. From these, the mean number of dogs per dog‐owning household and the human‐to‐dog ratio within the dog‐owning households were derived.

Because both surveys excluded households without dogs, a direct estimation of the national dog population was not possible. To address this, published estimates of household dog ownership were applied. For both Nigeria and Sierra Leone, the dog ownership prevalence was assumed to be 14%, based on an estimates from East Africa [24]. The mean number of dogs per household in the general population was estimated by multiplying the mean number of dogs per dog‐owning household by the assumed proportion of households owning dogs. This value was then extrapolated to the national level by multiplying by the estimated total number of households. Household counts were derived by dividing national population estimates by the average household size.

## 3. Statistical Analyses

### 3.1. Variable Selection

We designed a directed acyclic graph (DAG) to represent hypothesised causal relationships between socioeconomic, demographic, geographic and dog‐management factors and ownership of FRD (Figure 1), using the dagitty Version 0.34 and ggdag Version 0.2.13 packages in the RStudio environment [25–27]. Owner understanding of rabies was estimated using responses to binary rabies knowledge questions and an ordinal question about rabies host species and the literacy level. Household poverty was considered an upstream determinant influencing education. Education subsequently affected both occupation and owner knowledge regarding dog management practices. Urban/rural residences had multiple independent pathways of influence affecting occupation, the primary use of dogs, access to veterinary services and owner knowledge. Distance to the nearest road was modelled as a proxy for remoteness, influencing urban/rural residence and veterinary access. Household poverty also directly influenced access to veterinary services and FRD ownership, reflecting economic constraints on containment infrastructure and land availability. Given likely unmeasured confounding between poverty and urban–rural residence (e.g., shared regional infrastructure factors), we represented this relationship with a bidirected edge rather than a direct causal arrow. The primary use of dogs was influenced by occupation, urban–rural residence, and dog breed. Owner knowledge and access to veterinary services directly influenced FRD ownership. Dog breed directly influenced both primary use and free‐roaming ownership. The primary use of dogs mediated the effects of occupation, urban/rural residence and breed on FRD ownership. Dog vaccination status was modelled as a consequence of owner knowledge and access to veterinary services rather than as a determinant of FRD ownership, reflecting the assumption that better‐informed or better‐resourced owners are more likely to vaccinate regardless of confinement status. The history of dog bites was modelled as a downstream consequence of FRD ownership, given that roaming dogs have greater uncontrolled contact with people and was therefore excluded from the adjustment set for the ownership outcome. We applied the backdoor criterion to identify a minimal sufficient adjustment set for estimating effects on FRD ownership, confirming that this set blocks all backdoor paths without conditioning on colliders; therefore, the dog‐use category, a collider on a path between breed and residence, was excluded from adjustment.

**Figure 1:**
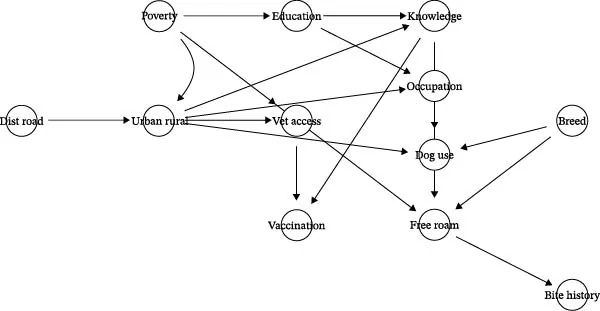
Directed acyclic graph (DAG) of hypothesised causal relationships among socioeconomic, demographic, geographic and dog‐management factors and FRD ownership. A minimal sufficient adjustment set for estimating effects on FRD ownership was identified using the backdoor criterion. Dog‐use category was excluded because it is a collider on the pathway between breed and residence (conditioning on it would introduce collider bias), and bite history was excluded because it is a downstream consequence of FRD ownership rather than a confounder.

### 3.2. Outcome of Interest

Our outcome of interest was the dog confinement status. Dogs were classified into three categories based on owner supervision and confinement: (1) fully confined, defined as dogs kept within the household premises and not allowed outside without owner supervision; (2) partially confined, defined as dogs that spent part of their time under supervision and the remainder unconfined and (3) FRDs, defined as dogs that did not spend time within a domicile. For the analysis, we created a binary outcome variable in which fully confined dogs were coded as 0, while partially confined dogs and FRDs were coded as 1.

This classification was based on the standard definition of FRDs, which included dogs that live and move with minimal or no direct human supervision or confinement [2, 28]. Importantly, these definitions include owned dogs that are allowed to roam freely or are not adequately confined by their owners. Therefore, partially confined dogs were grouped with FRDs because their unrestricted movement aligns with the operational definition used in the region. We constructed Bayesian logistic regression models to identify sociodemographic, dog ownership and environmental factors associated with the ownership of FRDs.

### 3.3. Multicollinearity Assessment for Nigeria and Sierra Leone

Before conducting the statistical analysis, we assessed multicollinearity using Pearson correlations, variance inflation factors (VIF) computed with the car package (Version 3.15) and the condition number for the model matrix [29]. Pearson correlation coefficients ranged from −0.149 to 0.247, indicating weak to negligible associations among covariates (Figure S1). The adjusted VIF values ranged from 1.05 to 1.29, well below the commonly used threshold of 5, indicating low levels of collinearity. We also calculated the condition number (*κ*) for the full model matrix, including interaction terms, and obtained a value of 35.3, well below the threshold of 100, indicating good numerical stability. Collectively, these diagnostics demonstrated that multicollinearity was not a concern in the fitted models despite the inclusion of multiple interaction terms. Before fitting our models, we assessed collinearity using bias‐corrected Cramér’s *V* for pairs of categorical predictors and generalised VIF for the full model. Predictors showed low collinearity overall (*V* = 0.00–0.48; adjusted VIF = 1.03–2.97), below common thresholds for concern (*V* < 0.5; VIF < 3) [30] (Figure S3).

### 3.4. Model Definition and Parameter Estimation for Nigeria

We specified five Bayesian logistic regression models using the brms package (Version 2.23.1) in R (Version 4.4.2), which used Stan for posterior inference via Markov chain Monte Carlo (MCMC) sampling [31, 32]. All models were fitted using Hamiltonian Monte Carlo with four independent MCMC chains, each run for 4000 iterations. The first 1000 iterations of each chain were discarded as warm‐up (burn‐in), yielding 3000 post‐warm‐up samples per chain (12,000 total posterior samples) for inference. Posterior inference was evaluated through visual inspection of trace plots to assess chain convergence, tail effective sample sizes (ESS), leave‐one‐out (LOO‐CV) [33] and posterior predictive checks (PPCs) to assess model fit.

For each model, we assumed that the outcome variable, ownership of an FRD, followed a Bernoulli distribution with probability parameter *p*. The probability of FRD ownership was modelled using a logit link function:

logitp=β0+ΣβkXk,

where *β*_0_ is the intercept and *β*_k_ represents the coefficient for the *k*th covariate, *X*_*k*_.

Model 1 included only fixed effects and was used to estimate the associations between the covariates and the probability of FRD ownership. Covariates included owner occupation, education level, rabies knowledge, awareness that rabid dogs can transmit the rabies virus, knowledge of susceptible hosts, residence type (urban versus rural), access to veterinary services, dog breed, vaccination status, proportion of people living in poverty and distance to the nearest road.

Given that dog ownership practices can vary across urban and rural settings and among different socio‐cultural groups in LMICs [14, 34], Model 2 extended Model 1 by including a state‐level spatial random intercept to account for unobserved geographic and sociodemographic heterogeneity across survey regions. This random intercept allowed for baseline differences in the probability of owning an FRD between states and the FCT while maintaining a common slope for all fixed effects. Model 3 further extended Model 2 by including interaction terms between residence type and education level, residence type and veterinary access, residence type and dog vaccination status and literacy level. These interactions were included to assess whether the associations between these variables and FRD ownership differed between urban and rural households. Model 4 added literacy as a main effect covariate (Model 4). Finally, Model 5 included an interaction term between literacy and education level to evaluate whether the association between educational attainment and FRD ownership varied according to the literacy status. Weakly informative priors were specified for all models. Fixed effects and intercepts were assigned normal (0, 2.5) priors, providing moderate regularisation on the logit scale, while the standard deviation of the regional random intercept was assigned a half‐Cauchy (0, 1) prior, allowing for between‐region variation while constraining estimates to positive values.

Model performance was evaluated using LOO‐CV [33], with expected log predictive density (elpd) used as the comparison criterion. PPCs were used to assess the model fit. Convergence was evaluated using trace plots, Rhat statistics (all <1.01), and tail ESS (all >400).

### 3.5. Model Selection

Model comparison using LOO‐CV [33] indicated that Model 5, incorporating interactions between residence type and education level, residence type and veterinary access, residence type and dog vaccination status and literacy level and education level, provided the best predictive performance (elpd = 0.0). Compared with simpler models, Model 5 improved predictive performance by up to 20.4 elpd units (SE = 7.4), suggesting that factors associated with FRD ownership varied across urban and rural settings and according to literacy and educational attainment. All model parameters converged satisfactorily (Rhat = 1.00) and had adequate Tail_ESS = 5139−10185 (Figure S2). The PPCs indicated a good model fit. Posterior summaries were used to estimate parameter effects and 95% credible intervals (CrIs), with coefficients considered significant when their 95% CrIs excluded zero. Full model outputs are provided in Supporting File Figure S2.

### 3.6. Posterior Visualisation of Key Parameters

We used the marginaleffects package (Version 0.32.0) [35] to estimate and visualise how the probability of FRD ownership varied across levels of selected covariates and interaction terms while accounting for parameter uncertainty. A total of 500 posterior draws were used to generate predicted probabilities under different predictor scenarios and to quantify posterior uncertainty. Spatial random effects were extracted and visualised using ggplot2 (Version 4.0.3) to map spatial variation across study locations [36]. We created a panel of figures using the patchwork package (Version 1.3.2) [37].

### 3.7. Model Definition and Parameter Estimation for Sierra Leone

We specified four Bayesian logistic regression models using the brms package (Version 2.23.1) in R (Version 4.4.2), which used Stan for posterior inference via MCMC sampling. All models were fitted using Hamiltonian Monte Carlo with four independent MCMC chains, each run for 4000 iterations. The first 1000 iterations of each chain were discarded as warm‐up (burn‐in), yielding 3000 post‐warm‐up samples per chain (12,000 total posterior samples) for inference.

Posterior inference was evaluated through visual inspection of trace plots to assess chain convergence, ESS, LOO‐CV for model comparison and PPCs to assess model fit.

For each model, we assumed that the binary outcome variable indicating FRD ownership followed a Bernoulli distribution with probability parameter *p*. The probability of FRD ownership was modelled using a logit link function:

logitp=β0+ΣβkXk,

where *β*_0_ was the intercept and *β_k_* represented the coefficient for the *k*th covariate, *X*_*k*_.

First, we fitted a baseline model (Model 1) containing only fixed effects to estimate the associations between predictor variables and FRD ownership. Covariates included owner occupation, education level, rabies knowledge, awareness that rabid dogs can transmit rabies virus, knowledge of susceptible hosts, residence type (urban versus rural), veterinary access (yes, no, or unsure), dog breed, dog vaccination status, proportion of people living in poverty, distance to the nearest road and human population density derived from a gridded population raster for Sierra Leone [38]. The distance to the nearest road (km) was log‐transformed after flooring values ≤0–0.05 km and subsequently standardised before inclusion in the model because of strong right‐skewness in the raw distribution.

Model 2 extended Model 1 by including a province‐level random intercept to account for unobserved geographic and sociodemographic heterogeneity across survey provinces. This random effect allowed baseline probabilities of FRD ownership to vary among provinces while maintaining common fixed‐effect slopes.

Model 3 further extended Model 2 by incorporating interaction terms between the education level and residence type, veterinary access and residence type and dog vaccination status and residence type. These interactions were included to assess whether associations between owner education, access to veterinary services, dog vaccination status and FRD ownership differed between urban and rural households.

Model 4 extended Model 3 by including an interaction between human population density and residence type (urban versus rural) to evaluate whether the association between population density and FRD ownership differed across urban and rural settings.

All fixed‐effect coefficients were assigned weakly informative normal (0, 2.5) priors, allowing the data to drive inference while providing modest regularisation. For the standard deviation of the province‐level random intercept, we evaluated both an exponential (1) prior and a half‐Cauchy (0, 1) prior. Both priors constrain the standard deviation to positive values while allowing for between‐province heterogeneity.

Model performance was assessed using LOO‐CV, with elpd used as the model comparison criterion. PPCs were used to assess the model fit to the observed data (Figure S4). Convergence was evaluated using trace plots, Rhat statistics (all <1.01) and Tail_ESS >400. The full model output, including parameter estimates, posterior summaries and Rhat values, is provided in Supporting File Figure S5.

### 3.8. Model Selection

Model comparison using LOO [33] showed that the fixed‐effects‐only model (Model 1) had an elpd difference of −48.2 (SE_diff_ = 11.0, *p*_worse_ = 1.00) relative to the best‐fitting model. The model including a province‐level random intercept (Model 2) had an elpd difference of −28.8 (SE_diff_ = 9.4, *p*_worse_ = 1.00). The model incorporating interaction terms between education level and residence type, veterinary access and residence type and dog vaccination status and residence type (Model 3) had an elpd difference of −8.8 (SE_diff_ = 3.6, *p*_worse_ = 0.99). The model that further included an interaction between residence type and human population density (Model 4) had an elpd difference of 0.0 (SE_diff_ = 0.0, *p*_worse_ = NA), indicating that it was the best‐fitting model and was therefore selected for inference.

All model parameters demonstrated satisfactory convergence (Rhat = 1.00) and adequate Tail_ESS = 6671–9029, substantially exceeding the recommended minimum threshold of 400 and indicating reliable MCMC sampling and posterior estimation (Figure S5). Trace plots (Figure S6) showed no evidence of poor mixing or erratic sampling behaviour. Furthermore, PPCs (Figure S7) demonstrated good agreement between the observed and predicted data, indicating an adequate model fit. Regression coefficients were considered significant when their 95% CrIs excluded zero.

### 3.9. Posterior Visualisation of Key Parameters and Spatial Prediction

We used the marginal effects package (Version 0.32.0) [35] to examine how the probability of FRD ownership varied across levels of selected covariates and interaction terms while accounting for uncertainty in parameter estimates. Posterior draws were used to visualise predicted effects under different predictor scenarios and to quantify posterior uncertainty.

We generated posterior predictions of FRD ownership across provinces using the brms package (Version 2.23.1) [31] and created a panel of figures using the patchwork package (Version 1.3.2) [37]. A standardised prediction dataset was constructed in which most covariates were held constant (occupation = student, rabies awareness = yes, rabies virus transmission knowledge = true, dog use = pet, education level = primary, residence type = rural, dog breed = mixed, vaccination status = vaccinated and veterinary access = no), while province was allowed to vary. Continuous covariates were fixed at their sample means.

Posterior predictive distributions were generated using the posterior_epred() function in two forms: (1) province‐specific predictions that included random intercepts and (2) population‐average predictions that excluded random effects. Spatial predictions were visualised as choropleth maps using the sf package (Version 1.0‐11) [39] and ggplot2 (Version 4.0.3) [36].

## 4. Results

### 4.1. Demographic Characteristics of Survey Participants and Dog Ownership Practices

Tables 1 and 2 summarise the demographic characteristics and dog management practices of dog‐owning households. In Nigeria, 59.7% (2485/4161) of households owned one or more FRDs, compared with 78.8% (2015/2558) in Sierra Leone.

**Table 1:** Demographic characteristics and dog ownership practices by dog confinement status among dog‐owning households in Nigeria, June and December 2022.

Characteristic	Confined *N* = 1677	Not confined/partial confinement *N* = 2485
Gender		
Female	643 (38%)	1135 (46%)
Male	1034 (62%)	1350 (54%)
Level of education		
None	127 (7.6%)	427 (17%)
Primary	155 (9.2%)	292 (12%)
Secondary	513 (31%)	1020 (41%)
Tertiary	882 (53%)	746 (30%)
Occupation		
Civil servant	434 (26%)	345 (14%)
Student	541 (32%)	734 (30%)
Private/NGO	702 (42%)	1406 (57%)
Is there any livestock officer or veterinary establishment in your location?		
Do not know	14 (0.8%)	66 (2.7%)
No	532 (32%)	1308 (53%)
Yes	1131 (67%)	1111 (45%)
Dog breed		
Indigenous	585 (34.9%)	1258 (50.6%)
Mixed	1092 (65.1%)	1227 (49.4%)
Has your dog been vaccinated against rabies in the last 12 months?		
Yes	460 (27%)	1509 (61%)
No	1217 (73%)	976 (39%)
Have you heard about rabies before?		
No	306 (18.3%)	951 (38.3%)
Yes	1371 (81.8%)	1534 (61.7%)
To your knowledge, have you or any members of your household been bitten by a dog in the last 2 years?		
Do not know	337 (20%)	513 (21%)
No	1129 (67%)	1632 (66%)
Yes	211 (13%)	340 (14%)
If you or any member of your family were ever bitten by a dog which method of treatment, would you prefer?		
Contemporary medicine	1320 (79%)	1730 (70%)
Traditional remedy	357 (21%)	755 (30%)
Rabies can infect the following?		
Do not know	228 (14%)	578 (23%)
Only human or animals	432 (26%)	741 (30%)
Humans and animals	1017 (61%)	1166 (47%)
State		
Kaduna	671 (40%)	579 (23%)
Anambra	215 (13%)	625 (25%)
Abuja	701 (42%)	971 (39%)
Enugu	90 (5.4%)	310 (12%)
Residence type		
Rural	822 (49%)	1508 (61%)
Urban	855 (51%)	977 (39%)

**Table 2:** Demographic characteristics and dog ownership practices by dog confinement status among dog‐owning households in Sierra Leone, July 2022–December 2023.

Characteristic	Confined *N* = 543	Not confined/partial confinement *N* = 2015
Gender		
Male	335 (62%)	1168 (58%)
Female	208 (38%)	847 (42%)
Level of education		
None	75 (14%)	751 (37%)
Primary	61 (11%)	331 (16%)
Secondary	236 (43%)	591 (29%)
Tertiary	171 (31%)	342 (17%)
Occupation		
Private/artisan	113 (21%)	284 (14%)
Student	123 (23%)	380 (19%)
Civil servant	307 (57%)	1351 (67%)
Have you heard about rabies before?		
Yes	155 (28.5%)	867 (43%)
No	388 (71.5%)	1139 (56.5%)
Rabies can infect the following?		
Humans and animals	—	—
Only humans/only animals	—	—
I do not know	—	—
If you or any member of your family were ever bitten by a dog which method of treatment, would you prefer?		
Traditional treatment	91 (17%)	691 (34%)
Contemporary medicine	452 (83%)	1324 (66%)
Province		
Eastern	163 (30%)	1004 (50%)
Southern	345 (64%)	835 (41%)
Western Area	17 (3.1%)	49 (2.4%)
North–Western	18 (3.3%)	127 (6.3%)
Residence type		
Urban	216 (40%)	266 (13%)
Rural	327 (60%)	1749 (87%)
Is there any livestock officer or veterinary establishment in your location?		
Yes	196 (36%)	308 (15%)
No	207 (38%)	1296 (64%)
I do not know	140 (26%)	411 (20%)
Breed of dog		
Crossed	493 (91%)	1957 (97%)
Indigenous	50 (9.2%)	58 (2.9%)
Has your dog been vaccinated against rabies in the last 12 months?		
Yes	144 (27%)	214 (11%)
No	399 (73%)	1801 (89%)
To your knowledge, have you or any members of your household been bitten by a dog in the last 2 years?		
Yes	31 (5.7%)	93 (4.6%)
No	442 (81%)	1628 (81%)
I do not know	70 (13%)	294 (15%)

To obtain nationally representative estimates, we applied post‐stratification weights based on national rural–urban population distributions. In Nigeria, 44.97% of the population resides in rural areas and 55.03% in urban areas, whereas in Sierra Leone, 55.22% resides in rural areas and 44.78% in urban areas, according to the World Bank and United Nations population estimates (theglobaleconomy.com). Stratum‐specific prevalences of FRD ownership (64.7% rural and 53.3% urban in Nigeria; 84.2% rural and 55.2% urban in Sierra Leone) were weighted using these population proportions, yielding estimated national prevalences of 58.5% in Nigeria and 71.2% in Sierra Leone.

The weighted national prevalence was calculated as follows:

Pw=Pr×Wr+Pu×Wu,

where *P*_*r*_ and *P*_*u*_ represent the prevalences of FRD ownership in rural and urban areas, respectively, and *W*_*r*_ and *W*_*u*_ represented the corresponding national rural and urban population proportions.

### 4.2. Dog Population

In Nigeria, 4162 dog‐owning households reported 9444 dogs and 26,155 humans. This corresponded to a mean of 2.27 dogs per dog‐owning household and an average household size of 6.29 persons. The human‐to‐dog ratio within dog‐owning households was 2.77:1. Assuming a national population of ~220 million and an average household size of 5.5–6.0 persons, the total number of households in Nigeria was estimated at 36.7–40.0 million. Applying a dog ownership rate of 14%, the number of dog‐owning households was estimated at 5.1–5.6 million. The mean number of dogs per household across the general population was estimated at 0.318, yielding an estimated national dog population of 11.7–12.7 million.

In Sierra Leone, 2558 dog‐owning households reported 8791 dogs and 24,166 humans. This corresponded to a mean of 3.44 dogs per dog‐owning household and an average household size of 9.45 persons. The human‐to‐dog ratio within dog‐owning households was 2.75:1. Assuming a national population of ~8.3 million and an average household size of six persons, the total number of households was estimated at 1.38 million. Applying a dog ownership rate of 14%, the number of dog‐owning households was estimated at 193,000. The mean number of dogs per household across the general population was estimated at 0.482, yielding an estimated national dog population of 666,000.

### 4.3. Factors Associated With the Ownership of FRDs in Nigeria

Our model estimates suggested that the likelihood of ownership of FRDs was lower among respondents whose dogs were vaccinated against rabies (*β* = −1.17, 95% CrI [−1.39, −0.95]), those who had heard about rabies (*β* = −0.41, 95% CrI [−0.61, −0.21]) and was higher with increased household poverty (*β* = 0.28, 95% CrI [0.10, 0.45]). Urban populations with tertiary education were significantly less likely to own FRDs (*β* = −1.18, 95% CrI [−1.76, −0.60]), while owners of mixed‐breed dogs were associated with lower ownership (*β* = −0.87, 95% CrI [−1.03, −0.71]) (Figure S2).

Predicted probability (covariates at modal values) showed FRD ownership was highest among respondents with no formal education, declining with education in rural areas but rising again at the tertiary level in urban areas. For veterinary access, ‘no services’ and ‘unsure’ responses were associated with higher FRD ownership in rural areas, whereas in urban areas, ‘no access’ showed the highest and ‘unsure’ the lowest estimates (Figure 2). The FRD ownership was higher among owners of indigenous versus mixed‐breed dogs and among owners of unvaccinated versus vaccinated dogs in both settings (Figure 3). Respondents unaware of rabies had a higher predicted likelihood of FRD ownership than those who were aware, while beliefs about rabies virus transmission by rabid dogs showed no clear effect, with overlapping CrIs (Figure 4). The FRD ownership showed a strong positive relationship with the poverty level, rising steadily from low to high poverty, while road distance had a negligible, near‐flat effect that widened in uncertainty at greater distances (Figure 5). Spatial random effects indicated lower predicted FRD ownership in Kaduna and FCT and higher predicted ownership in Anambra and Enugu (Figure 6).

**Figure 2:**
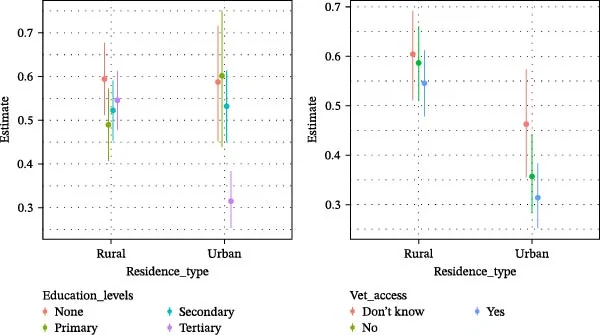
Posterior predicted probabilities showed that FRD ownership was highest among respondents with no formal education. The probability declined with increasing education in rural areas but increased at the tertiary level in urban areas (left panel). Limited or uncertain veterinary access was generally associated with higher FRD ownership, particularly in rural areas (right panel).

**Figure 3:**
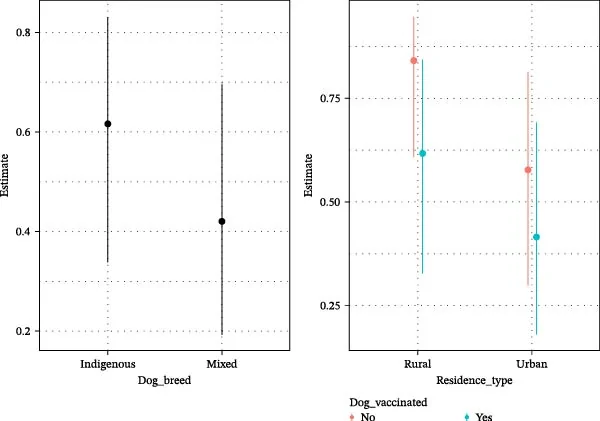
Posterior predicted probabilities (holding other covariates at their modal values) showed that FRD ownership was higher among owners of indigenous dogs than among owners of mixed‐breed dogs. Households with unvaccinated dogs were also more likely to own FRDs than those with vaccinated dogs in both rural and urban areas.

**Figure 4:**
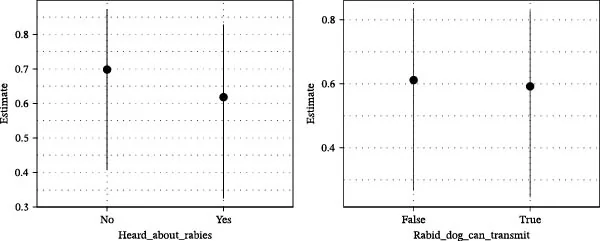
Posterior predictions (holding other covariates at modal values) showed modest effects of rabies knowledge on FRD. Respondents who had not heard about rabies showed a higher estimated likelihood of FRD ownership than those who had (left panel). Beliefs about whether rabid dogs can transmit rabies virus showed little difference between groups, with overlapping credible intervals indicating no clear effect (right panel).

**Figure 5:**
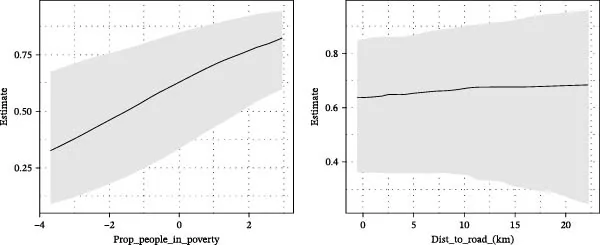
Posterior predictions (holding other covariates at their mean values) suggested a strong positive relationship between poverty proportion and FRD ownership, with the predicted likelihood rising steadily from low to high poverty levels; uncertainty was wide overall, particularly at lower poverty levels (left panel). Distance to a road (km) showed a negligible, near‐flat effect on FRD ownership, though uncertainty widened at greater distances (right panel).

**Figure 6:**
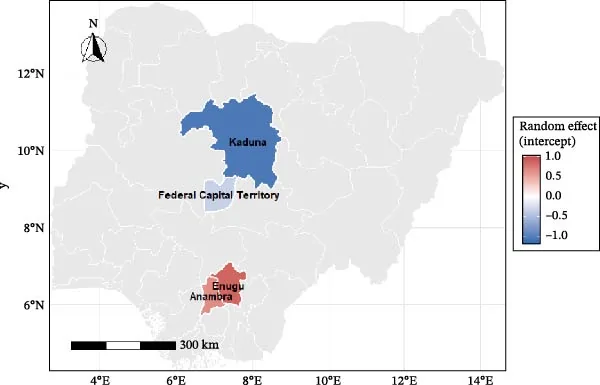
Spatial random effects for FRD ownership among dog‐owning households in Nigeria. Negative effects (blue) in Kaduna and FCT indicate lower predicted likelihood of keeping an FRD; positive effects (red) in Anambra and Enugu indicate higher predicted likelihood.

### 4.4. Factors Associated With the Ownership of FRD in Sierra Leone

Our model estimates suggested that the likelihood of ownership of FRDs was significantly reduced among respondents who understood that rabid dogs can transmit rabies virus (*β* = −0.37, 95% CrI [−0.66, −0.09]) and those with primary (*β* = −0.56, 95% CrI [−1.04, −0.08]), secondary (*β* = −0.99, 95% CrI [−1.40, −0.60]) and tertiary levels of education (*β* = −1.66, 95% CrI [−2.10, −1.22]). Dog vaccination status (*β* = −0.89, 95% CrI [−1.26, −0.52]), veterinary access (*β* = −0.63, 95% CrI [−1.10, −0.18]), mixed breed dogs (*β* = −1.27, 95% CI [−1.74, −0.79]) and human population density (*β* = −1.43, 95% CrI [−2.54, −0.32]) were all associated with reduced likelihood of FRD ownership. Conversely, respondents who were uncertain about which host rabies virus affects were more likely to own an FRD (*β* = 0.63, 95% CrI [0.23, 1.04]). Urban residents showed increased ownership likelihood with veterinary access (*β* = 1.74, 95% CrI [1.10, 2.40]), greater population density (*β* = 1.52, 95% CrI [0.48, 2.57]), vaccinated dogs (*β* = 1.03, 95% CrI [0.37, 1.70]) and yes to veterinary access (*β* = 1.30, 95% CrI [0.57, 2.04]) (Figure S5). Posterior estimates (covariates held at modal values for categorical and mean values for continuous predictors) showed no significant difference in FRD ownership between respondents who had heard of rabies and those who had not. Respondents who believed rabid dog bites cannot transmit rabies virus, or who did not know which species rabies virus affects, were more likely to own FRDs, and occupational status showed minimal variation across groups except for privately employed respondents, who had the highest probability (Figure 7). The FRD ownership was highest among respondents with no formal education in both urban and rural areas, while urban respondents with secondary education were less likely to own an FRD. In rural areas, respondents reporting no veterinary services or being unaware of any were more likely to own an FRD than those with access, while in urban areas, those reporting no veterinary services were more likely to own a FRD (Figure 8). There was a strong interaction between human population density and residence type on FRD ownership: rural households had high predicted ownership at low density, declining sharply as density increased, while urban households remained comparatively stable across the gradient. The FRD ownership also decreased with the increasing distance from roads (Figure 9). Additionally, FRD ownership was higher among respondents with unvaccinated dogs than vaccinated dogs and among those with indigenous dogs rather than mixed‐breed dogs, with spatial variation showing substantially higher ownership estimates in northern regions than southern regions. Right‐skewed distributions and wide CrIs across all regions reflected substantial uncertainty and data sparsity (Figure 10). Posterior distributions revealed spatial variation in the ownership of FRDs, with northern regions showing substantially higher estimates than southern regions (Figure 11). The full model output, including point estimates and Rhat values, is provided in the Supporting File Figure S5.

**Figure 7:**
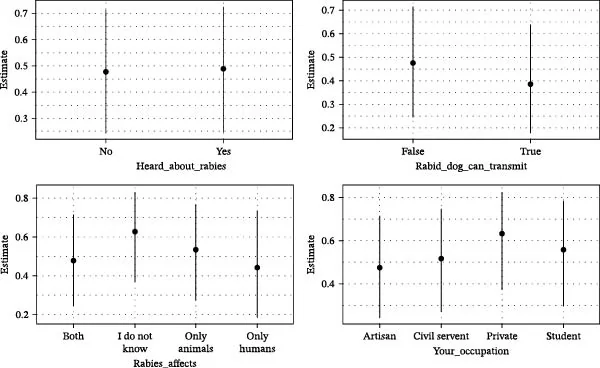
Posterior estimates from our model (holding other covariates at modal values) suggested that there was no significant difference in the probability of owing an FRD dog among respondents who have heard about rabies before and those who have not heard about rabies (top left panel). Respondents who believed rabid dog bites cannot transmit rabies virus (top right panel), or who did not know which species rabies virus affects (bottom left panel), were more likely to own FRDs. Occupational status showed minimal variation, with all groups having similar estimates of FRD ownership except privately employed individual, who had the highest probability (bottom right panel).

**Figure 8:**
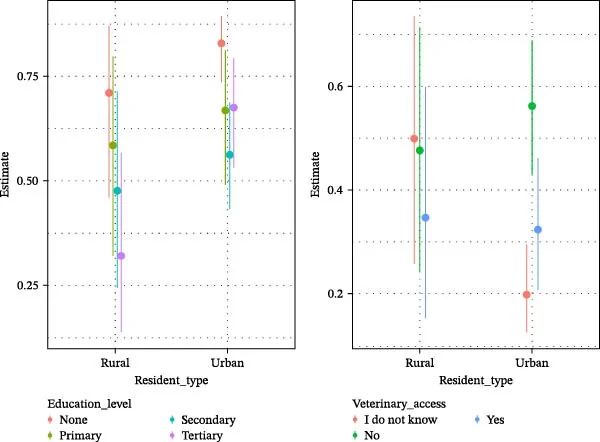
Posterior estimates from our model (holding other covariates at modal values) demonstrated that the likelihood of owning an FRD was highest among respondents with no formal education in both urban and rural areas, while in urban areas, respondents with secondary level of education were less likely to own an FRD (first panel). Respondents in rural areas who indicated that no veterinary services existed in their location and those who indicated they were unaware of the existence of one were more likely to own an FRD compared to those who indicated that a veterinary existed in their location, while in urban areas, those who indicated that no veterinary services existed in their location were more likely to own an FRD dog (second panel).

**Figure 9:**
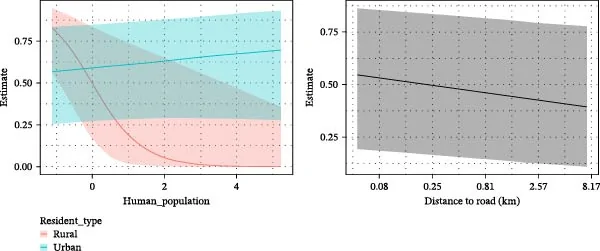
Posterior marginal effect estimates from our model showed a strong interaction between human population density and resident type on FRD ownership. Rural households had high predicted ownership probability at low density (~0.82), declining sharply to near zero (~0.02–0.05) as population density increased, while urban households remained comparatively stable (~0.57–0.70) across the gradient (left panel). Similarly, the ownership of FRD decreased with increasing distance from roads, from ~0.55 near roads to ~0.39 at ~8 km. Shaded areas (95% credible intervals) indicated substantial uncertainty, particularly at the extremes of each predictor’s range (right panel).

**Figure 10:**
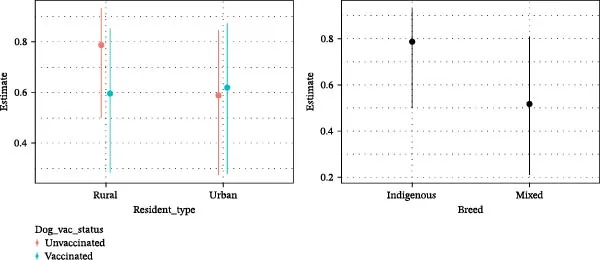
Posterior estimates from our model (holding other covariates at modal values) demonstrated that the likelihood of owning an FRD was highest among respondents with unvaccinated dogs compared to those with vaccinated dogs (first panel). Similarly, respondents with indigenous dogs were more likely to own FRDs compared to those with mixed‐breed dogs (second panel).

**Figure 11:**
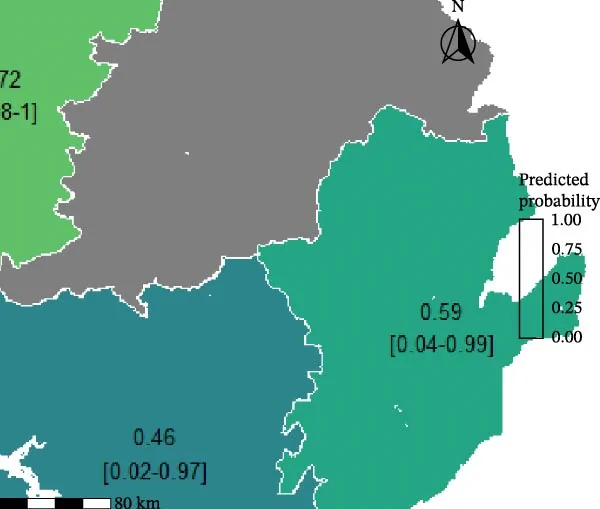
Posterior distributions revealed spatial variation in ownership of FRDs, with northern regions showing substantially higher estimates than southern regions. Northern areas had median probabilities of 0.82 (Northwestern; 95% CrI: 0.08–0.996) and 0.64 (Eastern; 95% CrI: 0.04–0.988), compared to southern areas at 0.43 (Southern; 95% CrI: 0.016–0.973) and 0.49 (Western Area; 95% CrI: 0.020–0.978). Wide credible intervals across all regions indicated substantial uncertainty, particularly in southern regions with intervals spanning near‐zero to >0.97. Right‐skewed posterior distributions were evident, as mean estimates (Northwestern: 0.72; Eastern: 0.59; Southern: 0.46; Western Area: 0.49) consistently fell below medians, reflecting data sparsity and heterogeneity. The grey area is the only province where the survey was not conducted.

## 5. Discussion

### 5.1. Summary of Findings

Measures aimed at controlling FRDs and reducing associated disease risks must be underpinned by an understanding of the factors that influence owners to allow their dogs to roam freely. We conducted a cross‐sectional study of more than 6000 dog‐owning households in Nigeria and Sierra Leone to identify factors associated with FRD ownership. Our findings provide evidence to inform policies and interventions aimed at improving FRD management. Education level, rabies knowledge, residence type (urban versus rural), access to veterinary services and dog breed were significantly associated with FRD ownership. Based on a human‐to‐dog ratio of 2.77:1, we estimated a dog population of ~11.7–12.7 million in Nigeria. In Sierra Leone, a human‐to‐dog ratio of 2.75:1 yielded an estimated dog population of 666,000. These estimates provide important evidence to support rabies control planning and the implementation of mass dog vaccination programs in both countries.

### 5.2. Estimated Dog Populations and Vaccination Targets

Our study estimated Nigeria’s dog population at 11.7–12.7 million based on a human‐to‐dog ratio of 2.77:1. This estimate aligns closely with a recent scoping review that reported 12,969,368 dogs (95% CI: 12,320,900−13,617,836) [40]. Given that a minimum vaccination coverage of 70% is recommended to interrupt rabies virus transmission [41], achieving herd immunity in Nigeria would require vaccinating ~9.1 million dogs based on the upper population estimate of 13.0 million. In Sierra Leone, we estimated a dog population of ~666,000, requiring the vaccination of about 466,200 dogs to achieve the recommended 70% coverage threshold. However, the 70% threshold represents vaccination coverage at a specific point in time rather than the cumulative number of vaccinations administered over an extended period. Dog populations are dynamic, with births, mortality, population turnover and cross‐border movement of dogs continually altering the population structure [42, 43]. Consequently, vaccination coverage achieved during a campaign progressively declines as unvaccinated dogs enter the population and previously vaccinated dogs die or lose protective immunity. Achieving and maintaining 70% vaccination coverage therefore requires the rapid and efficient implementation of mass dog vaccination campaigns at regular intervals rather than delivering an equivalent cumulative number of vaccinations over many years.

### 5.3. FRDs in Sierra Leone vs. Nigeria

Our study found a higher proportion of FRDs in Sierra Leone (78.8%; 95% CI: 77.1–80.3) than in Nigeria (59.7%; 95% CI: 58.2–61.2). Similarly, a survey in northern Thailand reported widespread dog ownership, with ~50% of households owning dogs and over 40% allowing unrestricted dog movement [44]. In Sierra Leone, the high prevalence of FRD ownership may partly reflect the long‐term effects of the civil war and subsequent socioeconomic instability. War‐related displacement and economic hardship contributed to widespread dog abandonment [45], while prolonged disruption of public services weakened animal control and public health systems. Limited municipal capacity and competing public health priorities have further constrained dog population management efforts [46].

Human behaviour may also contribute to sustaining FRD populations in Sierra Leone. The feeding of FRDs is relatively common, with one study reporting that ~77% of respondents provided food to FRDs [47]. Although such practices may be motivated by compassion or cultural norms, they provide a reliable food source that supports FRD survival and reproduction [48]. In addition, FRDs frequently rely on household and market waste, particularly in urban and peri‐urban areas, facilitating their persistence within human settlements and increasing opportunities for human‐dog interactions and conflicts [13].

Although the prevalence of FRDs was lower in Nigeria than in Sierra Leone, it remains substantial. Previous studies suggested that FRD populations in Nigeria are sustained by inadequate confinement practices, widespread access to waste for scavenging and limited access to veterinary services such as vaccination and sterilisation [8, 13]. The large FRD population, combined with low rabies vaccination coverage, presents a major challenge to rabies elimination efforts in both countries.

Access to veterinary services was also an important predictor of FRD ownership. Our model estimates indicated that, in both rural and urban settings, the probability of owning FRDs was greater among households reporting no access or uncertain access to veterinary services. In Sierra Leone, this finding may reflect severe limitations in veterinary infrastructure, with fewer than five veterinarians serving a population of several million people [49, 50]. Limited veterinary capacity restricts access to essential services, including rabies vaccination, sterilisation and treatment of animal diseases [45]. Consequently, opportunities for responsible dog management are reduced, contributing to uncontrolled breeding and increasing the risk of zoonotic disease transmission.

Collectively, these findings supported integrated approaches to FRD management that combine veterinary capacity building, mass dog vaccination, community education, improved waste management and humane dog population management strategies, including sterilisation where feasible. Such interventions, implemented within a coordinated One Health framework, could help reduce FRD populations and support sustainable rabies prevention and control in both countries.

### 5.4. Dog Vaccination Status and Residence Type

Households with unvaccinated dogs were more likely to allow them to roam freely than households with vaccinated dogs. This association likely reflects differences in owner awareness and management practices rather than a direct effect of vaccination, as owners who recognise rabies risks may be more likely to vaccinate and confine their dogs [51].

Rural households were also more likely to own FRDs than urban households, consistent with findings from Uganda and other LMICs [52]. This pattern may reflect differences in dog management and environmental conditions, including greater access to open spaces, food sources from poorly managed waste, fewer secure barriers and weaker enforcement of responsible dog ownership practices in rural areas [4, 52–55]. Together, these findings highlight the need for integrated interventions that combine community education, improved access to veterinary services and vaccination and better waste management to reduce FRD populations and support rabies control.

### 5.5. Spatial Variation and Socioeconomic Drivers of FRD Ownership

We observed spatial variation in FRD ownership. In Nigeria, respondents in Anambra and Enugu had a higher predicted probability of owning FRDs than those in the FCT. These differences may reflect regional variation in cultural attitudes and dog ownership practices [24, 56]. The lower probability of FRD ownership in the FCT may also be linked to its higher level of socioeconomic development, urbanisation and more structured infrastructure, which may favour more restricted dog management practices. Consistent with this explanation, our results showed that the probability of FRD ownership increased with household poverty in Nigeria.

In Sierra Leone, FRD ownership also varied spatially, with northern regions showing substantially higher predicted probabilities than southern regions. Previous studies have reported lower perceived importance of canine rabies vaccination among households in the northwestern region compared with the Eastern Province [57]. Regional differences in rabies awareness, attitudes toward dog management and access to animal health services may therefore contribute to the observed spatial variation in FRD ownership.

### 5.6. Education and Knowledge

Our study found that higher educational attainment was associated with a lower probability of FRD ownership in both rural and urban areas of Nigeria and Sierra Leone. This finding is consistent with studies from other LMICs, where education is associated with greater rabies awareness, more responsible dog ownership practices and higher vaccination uptake [8, 46, 58]. In settings where educational attainment remains relatively low, even basic education may substantially influence health‐related behaviours, including dog confinement, supervision and responses to dog bites [8, 46]. Interestingly, in Nigeria, primary education became significantly associated with reduced FRD ownership only when its interaction with literacy was included in the model. This finding suggests that formal education alone may not be sufficient to change behaviour and that the ability to understand and act on health information is also important. Previous studies have similarly shown that health literacy, media exposure and social environments influence whether education translates into safer health behaviours [59, 60].

Respondents with adequate rabies knowledge were also less likely to own FRDs, consistent with evidence from other rabies‐endemic LMIC settings where greater awareness was associated with more responsible dog management, increased vaccination uptake and reduced tolerance of FRDs [58, 61–65]. However, awareness of rabies does not necessarily translate into an actionable understanding of the viral transmission, clinical signs or prevention. Consequently, improving rabies control may require educational interventions that go beyond raising awareness and instead promote behaviour change and responsible dog ownership practices.

### 5.7. Dog Breed

Our model estimates showed that owners of indigenous dogs were more likely to allow their dogs to roam freely than owners of mixed‐breed dogs. In this context, mixed‐breed dogs refer to crosses between purebred dogs or between recognised breeds. Across many African settings, these dogs are often perceived as having higher economic value and may be purchased at substantially greater cost than indigenous dogs, which are commonly kept for guarding, hunting or community purposes rather than for breeding or companionship [66]. As a result, owners may be more likely to confine or closely supervise mixed‐breed dogs to reduce the risk of theft, injury, uncontrolled breeding or loss. This finding suggested that the perceived value of dogs may influence owner decisions regarding confinement and management practices.

## 6. Limitations

Our study has several limitations. First, the cross‐sectional design precludes the causal inference between the identified factors and FRD ownership. Second, the use of self‐reported data may have introduced recall, response and social desirability biases, potentially leading to over‐reporting of responsible practices such as dog confinement and vaccination. Third, because the study included only dog‐owning households, it may not fully capture the contribution of unowned and community dogs to the FRD population. Finally, some factors that may influence dog ownership practices, such as cultural attitudes toward dog management, owners’ motivations for allowing dogs to roam and household‐level socioeconomic conditions, were not measured directly. Although area‐level proxies for poverty and accessibility were included, residual confounding may remain. Despite these limitations, the study provided important insights to inform dog population management and rabies control strategies in Nigeria and Sierra Leone.

## 7. Conclusion

In conclusion, FRD ownership is common in both Nigeria and Sierra Leone and was influenced by sociodemographic, dog management and environmental factors. Targeted public education, improved access to veterinary services and rabies vaccination and community‐level interventions in rural and other high‐risk areas may help reduce FRD populations and strengthen rabies control efforts. Based on human‐to‐dog ratios of 2.77:1 in Nigeria and 2.75:1 in Sierra Leone, we estimated dog populations of 11.7–12.7 million and 666,000, respectively. These estimates provide valuable evidence for planning and implementing mass dog vaccination campaigns in both countries with an improvement in both animal welfare and public health .

## 8. Recommendations

These findings highlight the need for targeted public education, improved access to veterinary services and rabies vaccination and community‐level interventions in rural and high‐risk areas. Strengthened vaccination campaigns and humane dog population management strategies may help reduce FRD populations and support sustainable rabies control. The estimated dog populations can inform the planning and implementation of mass dog vaccination campaigns in both countries as well as other LMICs.

## Author Contributions

**Philip P. Mshelbwala:** conceptualisation, methodology, data curation, formal analysis, visualisation, original draft, writing – review and editing. **J. Scott Weese, Tasneem Imam and Charles E. Rupprecht:** review and editing. **Harish K. Tiwari:** administration. **Roland Suluku:** investigation, project administration, writing – review.

## Funding

The authors received no specific funding for this work.

## Conflicts of Interest

The authors declare no conflicts of interest.

## Supporting Information

Additional supporting information can be found online in the Supporting Information section.

## Supporting information


**Supporting Information** Supporting file showing the result of multicollinearity assessments, model outputs, convergence diagnosis and model check. Figure S1: Multicollinearity assessment of variables used in the model, indicating that all correlations ranged from −0.149 to 0.247, suggesting weak to negligible relationships. Figure S2: Model output including point estimates for Nigeria, Rhat and sufficient effective sample sizes (Tail_ESS ranging from 4830–16,94, all well above the recommended minimum of 400). Figure S3: Bias‐corrected Cramér’s *V* for categorical predictor pairs and generalised VIF for the full model showed low collinearity overall (V = 0.00–0.48; adjusted VIF = 1.03–2.97), below common thresholds for concern (V < 0.5; VIF < 3) [38]. Figure S4: Posterior predictive check result demonstrating good fitting of our Nigerian model (yrep) to the observed data (y). Figure S5: Model output including point estimates for Sierra Leone, including Rhat and sufficient effective sample sizes, demonstrating good model fit (4650–17,654). Figure S6: Trace plot showing good mixing of the chain for our Sierra Leone model. Figure S7: Posterior predictive check result demonstrating good fitting of model (yrep) to the observed data (y) for the Sierra Leone model.

## Data Availability

The data that support the findings of this study are available from the corresponding author upon reasonable request.
