# Supplementary material for: Sociodemographic and Dog‐Management Factors Associated With Free‐Roaming Dogs in Nigeria and Sierra Leone
Source: Transbound Emerg Dis. 2026 Sep 29;2026:8621173. doi: 10.1155/tbed/8621173 (PMC13624544; doi:10.1155/tbed/8621173)
Supplement: Supplementary file 1 [file TBED-2026-8621173-s001.docx]

Figure S1: Multicollinearity assessment of variables used in the model, indicating that all correlations ranged from −0.149 to 0.247, suggesting a weak to negligible relationships.

Figure S2: Model output including point estimates for Nigeria, Rhat and sufficient effective sample sizes (Tail_ESS ranging from 4,830–16,94, all well above the recommended minimum of 400).

Figure S3: Bias-corrected Cramér's V for categorical predictor pairs and generalised VIF for the full model showed low collinearity overall (V = 0.00–0.48; adjusted VIF = 1.03–2.97), below common thresholds for concern (V < 0.5; VIF < 3)[37]

Figure S4: Posterior predictive check result demonstrating good fitting of our Nigerian model(y_rep_) to the observed data(y)

Figure S5: Model output including point estimates for Sierra Leone, including Rhat and sufficient effective sample sizes, demonstrating good model fit (4,650 -17,654).

Figure S6: Trace plot showing good mixing of the chain for our Sierra Leone model

s7: Posterior predictive check result demonstrating good fitting of model(y_rep_) to the observed data(y) for Sierran Leone model
